# Effect of a Behavioral Intervention on Perpetrating and Experiencing Forced Sex Among South African Adolescents: A Secondary Analysis of a Cluster Randomized Trial

**DOI:** 10.1001/jamanetworkopen.2018.1213

**Published:** 2018-08-17

**Authors:** John Barton Jemmott, Ann O’Leary, Loretta Sweet Jemmott, Zolani Philemon Ngwane, Anne Marie Teitelman, Monde Blessing Makiwane, Scarlett L. Bellamy

**Affiliations:** 1 Annenberg School for Communication, Center for Health Behavior and Communication Research, University of Pennsylvania, Philadelphia; 2 University of Pennsylvania, Philadelphia; 3 Centers for Disease Control and Prevention, Atlanta, Georgia; 4 Drexel University, Philadelphia, Pennsylvania; 5 Haverford College, Haverford, Pennsylvania; 6 Human Sciences Research Council, Pretoria, South Africa

## Abstract

**Importance:**

Scant research has investigated interventions to reduce forced sexual intercourse among adolescents. The need for such interventions is especially great in South Africa, which has some of the highest rates of sexual assault in the world.

**Objectives:**

To determine whether an HIV/sexually transmitted disease risk-reduction intervention that reduced sexual risk behavior and sexually transmitted disease prevalence also reduced the perpetration and experience of forced sex among South African adolescents.

**Design, Setting, and Participants:**

A cluster randomized clinical trial, at schools located in a township and a semirural area, Eastern Cape Province, South Africa. Matched pairs of schools were randomly selected (9 of 17); of 1118 students in sixth grade at these 18 schools who had parent or guardian consent, 1057 (94%) were enrolled, and those not reporting forced sex perpetration before the intervention were included in the analyses (n = 1052). Post hoc secondary analysis of a cluster randomized clinical trial was performed, with baseline and 3-, 6-, 12-, 42-, and 54-month postintervention assessments between October 4, 2004, and June 30, 2010. Generalized estimating equation Poisson regression analyses adjusting for gender and clustering within schools were conducted between August 23, 2017, and April 30, 2018. Recruiters and data collectors, but not intervention facilitators, were blind to the participants’ intervention assignment.

**Interventions:**

Theory-based, culturally adapted, 6-session HIV/sexually transmitted disease risk-reduction intervention (Let Us Protect Our Future intervention) and attention-matched, chronic disease prevention control intervention implemented by specially trained man and woman cofacilitators from the community.

**Main Outcomes and Measures:**

Study outcomes for this secondary analysis (planned after the data were collected) are self-reports of perpetrating and experiencing forced vaginal intercourse.

**Results:**

Participants included 1052 adolescents (557 girls [53%]; mean [SD] age, 12.4 [1.2] years) reporting not perpetrating forced sex at baseline. Fewer intervention than control participants reported forced sex perpetration postintervention compared with the control group at 3 months (9 of 561 [2%] vs 20 of 491 [4%]; risk ratio [RR], 0.978; 95% CI, 0.959-0.997), 6 months (17 of 561 [3%] vs 35 of 491 [7%]; RR, 0.964; 95% CI, 0.941-0.988), 12 months (21 of 561 [4%] vs 42 of 491 [9%]; RR, 0.959; 95% CI, 0.934-0.985), 42 months (41 of 561 [7%] vs 56 of 491 [11%]; RR, 0.967; 95% CI, 0.937-0.998), and 54 months (52 of 561 [9%] vs 68 of 491 [14%]; RR, 0.964; 95% CI, 0.932-0.997).

**Conclusions and Relevance:**

In settings with high rates of sexual assault, the use of theory-based culturally adapted interventions with early adolescents may reduce rates of perpetrating and experiencing forced sex.

**Trial Registration:**

ClinicalTrials.gov Identifier: NCT00559403

## Key Points

**Question:**

Can a 12-hour theory-based behavioral intervention culturally adapted for students in sixth grade in South Africa reduce forced sexual intercourse perpetration among adolescents?

**Findings:**

In this secondary analysis of a cluster randomized clinical trial that included 1052 South African adolescents, the percentage reporting perpetration of forced sexual intercourse by 54 months postintervention was 9% in the intervention group and 14% in the control group, a significant difference.

**Meaning:**

In settings with high rates of sexual assault the use of theory-based, culturally adapted interventions to reduce the prevalence of forced sexual intercourse perpetration may be warranted.

## Teaser

This secondary analysis of a cluster randomized clinical trial evaluates whether an HIV/sexually transmitted disease (STD) risk-reduction intervention shown to reduce sexual risk behavior and STD prevalence among South African adolescents also reduced the perpetration and experience of forced sex.

## Introduction

The prevalence of forced sex is high in South Africa^1,2^ and throughout southern Africa generally.^3,4,5^ Although most sexual violence studies have focused on adults, recognition of the problem of sexual violence among adolescents is growing.^6^ Studies in the United States^7,8^ and Europe^9,10^ highlight the high rates of experienced forced sex among girls,^7,8^ but in southern Africa, high rates of experienced forced sex have been observed in both girls and boys.^3^ For instance, in a survey of school-going adolescents in 10 southern African countries, 20% of girls and 20% of boys reported experiencing forced sex, and 5% of girls and 12% of boys reported perpetrating forced sex.^3^ Forced sex experiences during adolescence can have deleterious consequences, including increased risk of depression,^11,12^ suicide ideation,^11,12,13^ substance use,^12,14^ early pregnancy,^12,13^ and sexually transmitted infections,^8^ including HIV.^15^ Developmentally, adolescence provides a unique opportunity to promote behaviors that prevent sexual violence over the life course because during this period gender role differentiation intensifies and children try out new ways of thinking and acting in intimate relationships.^16,17^

Reviews of the literature suggest that behavioral interventions can reduce intimate partner violence (IPV) among adolescents,^6,16,17^ but most studies have not examined sexual violence separate from other types of IPV (eg, physical and psychological violence). Some have examined the experience or perpetration of IPV, but not both, and few have examined effects of interventions on experience of forced sex among adolescent boys in low- or middle-income countries.^16^ Moreover, many studies had weak designs, short follow-up periods, or high attrition rates.^16^ A recent review^17^ asserted that only 3 primary prevention strategies have reduced sexual violence in rigorous outcome evaluations: Safe Dates,^18,19^ Shifting Boundaries,^20^ and funding associated with the 1994 Violence Against Women Act.^21^ In Safe Dates, a 14-school cluster randomized clinical trial (RCT) with 50% of 1566 students in eighth grade retained at 3-year follow-up, intervention participants experienced less forced sexual behavior than did no-treatment controls.^19^ In Shifting Boundaries, a 30-school cluster RCT on 2655 students in middle school randomized to a classroom-based, building-based, combined, or no intervention group, the building-only and combined intervention participants experienced less sexual violence than did controls at 6-month follow-up.^20^ Most trials of forced sex interventions have been conducted in the United States or other high-income countries, which has led to calls for rigorous intervention trials in low- and middle-income countries.^16,22^

This article reports secondary analyses of the efficacy of Let Us Protect Our Future, a theory-based, culturally appropriate, HIV risk-reduction intervention, in reducing the experience and perpetration of forced sex in a middle-income country, South Africa, where forced sex is exceptionally common.^23^ In a cluster RCT,^24^ 18 schools serving students in sixth grade in Eastern Cape Province, South Africa, were randomized to Let Us Protect Our Future or an attention-matched control group. The primary outcome analyses indicating the intervention reduced sexual risk behaviors,^24,25^ analyses of the mediation of its efficacy,^26^ and analyses indicating it reduced sexually transmitted disease (STD) prevalence among sexually experienced adolescents were reported elsewhere.^25^ The intervention was developed based on formative data to address not only sexual risk reduction but also gender issues and rape myth beliefs and included activities to reduce the risk of perpetrating and experiencing forced sex.^27^ We hypothesized that the interventions focused on reducing the risk of forced sex would translate into fewer adolescents reporting perpetrating forced sex or experiencing forced sex in the intervention group compared with the attention-matched control group.

## Methods

This article reports post hoc secondary analyses conducted between August 23, 2017, and April 30, 2018 of a cluster RCT. We followed the Consolidated Standards of Reporting Trials (CONSORT) reporting guideline. Institutional review board No. 8 at the University of Pennsylvania, the designated institutional review board under the federal wide assurances of the University of Pennsylvania and the University of Fort Hare, approved the study (trial protocol is available in the Supplement). Written parent or guardian permission and adolescent assent were required for participation. As reported elsewhere,^24^ we conducted the study in an urban township, Mdantsane, and a neighboring semirural settlement, Berlin, in Eastern Cape Province, South Africa. Schools serving students in sixth grade from the general population were eligible. Of 36 schools serving students in sixth grade in the catchment area, 1 serving children with learning disabilities was ineligible, leaving 35 eligible schools, all agreeing to participate. From 17 matched pairs of schools similar in numbers of students in sixth grade, classrooms, and classrooms with electricity, including 1 pair consisting of 3 schools, we randomly selected 9 pairs.

We used a cluster RCT design, reducing the potential for contamination between treatment groups that would be present were individuals randomized. We enrolled schools over 13 months beginning in October 4, 2004. The trial biostatistician identified computer-generated random number sequences to randomize, within pairs, 1 school to the HIV/STD risk-reduction intervention and 1 to the control group. The project director implemented the assignments. Recruiters, following a standardized scripted recruitment protocol, announced the study at the schools and distributed cover letters and parent or guardian permission forms to students in sixth grade. During recruitment, school personnel, potential participants, and recruiters were masked to the schools’ randomized intervention assignment. The nature of the intervention precluded masking the facilitators and participants to the group assignment during the interventions.

### Interventions

The Let Us Protect Our Future intervention was developed based on social cognitive theory^28^ and the theory of planned behavior,^29^ integrated with qualitative information from extensive formative research with the target population.^27^ It included 12 one-hour modules, with 2 modules delivered during each of 6 sessions on consecutive school days involving games, brainstorming, role-playing, group discussions, and comic workbooks with a series of characters and story lines. Although the intervention was primarily designed to reduce sexual risk behaviors, it included several features designed to address gender issues and rape myth beliefs relevant to perpetration and experience of forced sex. Formative research suggested that girls were in danger of sexual assault if they accepted drinks, snacks, gifts, or a taxi ride from a man, who might then see himself as entitled to have sex afterward; accordingly, the intervention included activities to reduce this risk.^27^ One was a doll activity to challenge negative attitudes toward women and sexual coercion. Participants used dolls with changeable clothing to express their views on how young women dress in music videos, work, and school and considered whether a woman’s clothing is a legitimate reason to infer that she has bad character and whether a girl who dresses sexy is asking for sex or deserves to be forced to have sex.

To increase participants’ skills and self-efficacy to avoid risky situations, we created the “Long Walk Home” in which participants identified risky situations and/or men they might encounter on their way to or from school. They traced the safest paths on a map and brainstormed strategies to reduce their risk of sexual coercion. Indeed, the scale measuring self-efficacy to avoid risky situations was a significant mediator of the intervention’s effects on abstinence; particularly among girls compared with boys.^26^ The “Stop, Think, and Act” activity reinforced refusal skills and impulse control beliefs. The “What Is a Relationship” activity reinforced pride in having a healthy relationship. The “Understanding Risky Situations” activity reinforced being aware of risky situations and how to plan to avoid them. The “Knowing and Setting Sexual Limits” activity helped the participants to be able to know and express their limits to avoid risky behaviors. Finally, participants practiced sex refusal by stomping a foot and saying “No!” Beyond these activities, the intervention was implemented in mixed-sex groups of 9 to 16 adolescents cofacilitated by a specially trained man and woman, and these facilitator pairs modeled egalitarian gender roles in delivering the intervention.

The 12-hour health promotion control intervention^30^ included activities similar to the HIV/STD risk-reduction intervention delivered over 6 sessions cofacilitated by a specially trained man and woman, targeting physical activity and fruit and vegetable consumption, behaviors linked to chronic diseases that are leading causes of death in South Africa.^31,32^ The interventions were pilot tested in English in Mdantsane, translated into Xhosa, back-translated from Xhosa to English, pilot tested in Xhosa in Mdantsane and Berlin, and delivered in Xhosa in the trial.

### Procedures

We enrolled in the trial students in sixth grade who completed the preintervention questionnaire and attended session 1 of the intervention. They completed immediate and 3-, 6-, and 12-month postintervention questionnaires by December 15, 2006. Intervention and data collection sessions were held at the students’ school. The initial informed assent and parent permission process covered activities through the 12-month follow-up.^24^ Accordingly, we located the students, then attending more than 200 secondary schools, and gave them parent or guardian permission forms and cover letters explaining the continuation of the trial and inviting their parents or guardians to a meeting at which they could ask questions about the follow-up study.

We began the 42-month data collection in April 19, 2008, and completed the 54-month data collection on June 30, 2010.^25^ As compensation, students received a notebook, a pen, and a pencil for the 3-month follow-up; a T-shirt for the 6-month follow-up; a backpack for the 12-month follow-up; an umbrella (for girls) or a cap (for boys) for the 42-month follow-up; and a jacket for the 54-month follow-up. We held the intervention and data collection sessions, except 42- and 54-month follow-ups, at the students’ schools during the extracurricular period at the end of the school day. We held the 42- and 54-month follow-ups on Saturdays at 1 of the 18 schools, a centrally located school with suitable plumbing facilities, and provided transportation to the sessions.

### Outcomes

Participants completed confidential questionnaires containing questions on forced sex before the intervention and 3, 6, 12, 42, and 54 months after the intervention administered by data collectors who were blind to participants’ intervention assignment. As noted elsewhere,^24^ we took several steps to increase the validity of self-reports; the questionnaires were written in Xhosa following translation and back-translation from English and were pilot tested with adolescents from the population.^24^ The forced sex outcomes in this secondary analysis were not described in the trial registration. We assessed forced sex at each assessment with measures whose reliability and validity has been established in previous studies,^33,34,35^ including pilot studies with Xhosa-speaking adolescents.^36,37^ We defined vaginal intercourse as “your penis in a girl’s vagina” (male version) or “a boy’s penis in your vagina” (female version). Binary variables were used to assess history of ever perpetrating and experiencing forced sexual intercourse, with responses coded 1 for respondents not reporting ever experiencing the event and 2 for those reporting ever experiencing the event. To assess perpetrated forced vaginal intercourse, participants were asked, “Have you ever had vaginal intercourse with someone who did not want to have sex with you?” To assess experienced forced vaginal intercourse, participants were asked, “Have you ever been forced to have vaginal intercourse against your will?”

### Statistical Analysis

The a priori unit of inference was the individual.^24^ A sample size calculation was performed to detect an effect of *d* = 0.25 SD^38^ on the a priori primary outcome, unprotected intercourse, adjusting for the expected variance inflation due to clustering.^39^ Assuming α = .05, a 2-tailed test, an intraclass correlation coefficient = 0.00864 based on unpublished pilot data, 20% attrition, and 1100 students in sixth grade enrolled in the trial from 16 schools with an average of 67 students in each school, the trial was estimated to have 80% power to detect *d* = 0.25 effect of the intervention.

The efficacy of the HIV/STD intervention compared with the control intervention on the forced sex outcomes was tested using generalized estimating equation Poisson regression models, adjusting for gender and students clustered within schools.^40,41^ Forced sex was operationalized as being present for each participant for any subsequent follow-up assessment after being initially reported (eg, incidence of ever reporting perpetrating or experiencing forced sex over the course of the study) and operationalized as being absent if and only if forced sex was never reported at any visit. The model was tested at each of the 5 postintervention assessments. Additional analyses tested the intervention effect on time-specific incidence adjusting for time. Robust standard errors were used and an exchangeable working correlation matrix was specified. Risk ratios^42^ and corresponding 95% confidence intervals are reported. Analyses of postintervention perpetration excluded 5 participants in the original sample who reported ever perpetrating forced sex at baseline, and analyses of postintervention experience of forced sex excluded 7 participants in the original sample who reported ever having such experience at baseline. In the analyses, participants (4 controls and 3 intervention participants) who were missing at all postintervention assessments were coded as not experiencing the event.

We conducted exploratory moderator analyses, using intervention condition × sex interactions, to test whether the effect of the intervention was significantly different in boys compared with girls, adjusting for the effects of intervention and sex. The significance criterion was set at α = .05, 2-tailed tests. All analyses were intention-to-treat analyses and completed using SAS, version 9 (SAS Institute Inc).

## Results

The Figure shows the flow of participating schools and adolescents through the trial. The participants included 1052 adolescents aged 9 to 18 years (557 girls [53%]; mean [SD] age, 12.4 [1.2] years) who did not report perpetrating forced sex at baseline. A total of 1045 (99%) returned for at least 1 follow-up, with no difference between the intervention (99%) and control condition (99%). Fewer intervention than control participants reported forced sex perpetration postintervention compared with the control group at 3 months (9 of 561 [2%] vs 20 of 491 [4%]; risk ratio [RR], 0.978; 95% CI, 0.959-0.997), 6 months (17 of 561 [3%] vs 35 of 491 [7%]; RR, 0.964; 95% CI, 0.941-0.988), 12 months (21 of 561 [4%] vs 42 of 491 [9%]; RR, 0.959; 95% CI, 0.934-0.985), 42 months (41 of 561 [7%] vs 56 of 491 [11%]; RR, 0.967; 95% CI, 0.937-0.998), and 54 months (52 of 561 [9%] vs 68 of 491 [14%]; RR, 0.964; 95% CI, 0.932-0.997). Table 1 shows that 557 girls (53%) and 495 boys (47%) participated; 79 participants (8%) resided in the rural settlement and the others resided in the urban township. Table 2 shows the incidence of participants reporting perpetrating and experiencing forced sex × intervention condition at each assessment. Table 3 shows the HIV/STD risk-reduction intervention reduced self-reported forced sex perpetration at each of the 5 postintervention assessments compared with the attention-matched control group. Similarly, in the analysis on incidence, the intervention’s effect in reducing forced sex perpetration was significant, adjusting for time.

**Figure.**
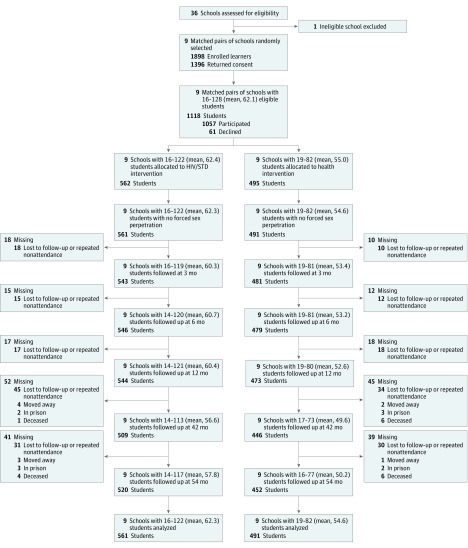
Flowchart of Participating Schools and Adolescents Through the Trial One school was ineligible because it exclusively served children who had learning disabilities, for whom the type of intervention planned was inappropriate. A total of 17 schools not randomly selected were excluded from participation. At 6 schools, there were too few classrooms to accommodate all of the students who had consent; accordingly, we randomly selected students as eligible from among those with consent, resulting in 278 students who were deemed ineligible. One student in the HIV/sexually transmitted disease (STD) intervention and 4 students in the health promotion intervention reported perpetrating forced sex at baseline.

**Table 1.** Sociodemographic Characteristics of Participating Schools and Students in Sixth Grade by Intervention Condition at Baseline, Mdantsane and Berlin, South Africa, 2004 to 2005

Characteristics at Baseline	HIV/STD Intervention	Health Control Intervention	Total
School			
No.	9	9	18
Rural, No.	2	2	4
Urban, No.	7	7	14
Classrooms, mean (SD), No.	9.7 (3.2)	8.9 (2.7)	9.3 (2.9)
Classrooms with electricity, mean (SD), No.	5.6 (5.8)	3.3 (3.8)	4.4 (4.9)
Students			
No.	561	491	1052
Female, No. (%)	306 (55)	251 (51)	557 (53)
Father present in household, No./total No. (%)	203/543 (37)	192/475 (40)	395/1018 (39)
Rural resident, No. (%)	40 (7)	39 (8)	79 (8)
Age, No. (%), y			
9-11	144 (26)	104 (21)	248 (24)
12-13	329 (59)	301 (61)	630 (60)
14-18	88 (16)	86 (18)	174 (17)

Abbreviation: STD, sexually transmitted disease.

**Table 2.** Empirical Distribution of Cumulative Incidence and Time-Specific Incidence of Self-reported Forced Sexual Intercourse Perpetration and Experiences by Intervention Condition and Postintervention Assessment Period Among Adolescents, Mdantsane and Berlin, South Africa, 2004 to 2010

Assessment Period	No./Total No. (%)			
	Cumulative Incidence		Time-Specific Incidence	
	HIV Risk-Reduction Intervention	Attention-Control Intervention	HIV Risk-Reduction Intervention	Attention-Control Intervention
Perpetrated forced sex, mo				
3	9/561 (2)	20/491 (4)	9/561 (2)	20/491 (4)
6	17/561 (3)	35/491 (7)	8/552 (1)	15/471 (3)
12	21/561 (4)	42/491 (9)	4/544 (1)	7/456 (2)
42	41/561 (7)	56/491 (11)	20/540 (4)	14/449 (3)
54	52/561 (9)	68/491 (14)	11/520 (2)	12/435 (3)
Experienced forced sex, mo				
3	13/559 (2)	21/491 (4)	13/559 (2)	21/491 (4)
6	22/559 (4)	33/491 (7)	9/546 (2)	12/470 (3)
12	30/559 (5)	47/491 (10)	8/537 (1)	14/458 (3)
42	50/559 (9)	67/491 (14)	20/529 (4)	20/444 (5)
54	72/559 (13)	82/491 (17)	22/509 (4)	15/424 (4)

**Table 3.** Generalized Estimating Equation Empirical Significance Tests, Risk Ratios for the Intervention Effect on Self-reported Forced Sexual Intercourse Perpetration and Experiences by Assessment Period Among Adolescents, Mdantsane and Berlin, South Africa, 2004 to 2010

Assessment Period, mo	Intervention Effect on Perpetrating Forced Sex (n = 1052)		Intervention Effect on Experiencing Forced Sex (n = 1050)	
	RR (95% CI)	*P* Value	RR (95% CI)	*P* Value
3	0.978 (0.959-0.997)	.02	0.983 (0.963-1.004)	.11
6	0.964 (0.941-0.988)	.004	0.977 (0.953-1.002)	.07
12	0.959 (0.934-0.985)	.002	0.966 (0.939-0.994)	.02
42	0.967 (0.937-0.998)	.04	0.963 (0.931-0.996)	.03
54	0.964 (0.932-0.997)	.03	0.972 (0.937-1.008)	.12
3-54	0.990 (0.982-0.999)	.02	0.992 (0.982-1.001)	.09

Abbreviation: RR, risk ratio.

Table 3 also shows that the HIV/STD risk-reduction intervention reduced the risk of experiencing forced sex at the 12- and 42-month postintervention assessments compared with the attention-matched control group, but not at 3, 6, or 54 months postintervention. However, in the analysis on incidence, the intervention’s effect in reducing forced-sex experience was not significant.

Table 4 shows reports of perpetrating and experiencing forced sex were significantly higher among boys compared with girls at each of the postintervention assessments, adjusting for the intervention’s effects in both the cumulative and time-specific incidence analyses. Also in Table 4, intervention condition × sex interactions on both forced sex perpetration and experience were significant at 3-, 6-, and 12-month follow-up, but not at 42- or 54-month follow-up, indicating the intervention was more efficacious in reducing forced sex among boys than among girls. Among boys, the effect on perpetration of forced sex was significant at 3-month follow-up (RR, 0.952; 95% CI, 0.917-0.988), 6-month follow-up (RR, 0.934; 95% CI, 0.892-0.979), and 12-month follow-up (RR, 0.924; 95% CI, 0.879-0.972) and the effect on forced sex experience was also significant at 3-month follow-up (RR, 0.959; 95% CI, 0.921-0.999), 6-month follow-up (RR, 0.948; 95% CI, 0.904-0.995), and 12-month follow-up (RR, 0.932; 95% CI, 0.883-0.983). In contrast, among girls, effects were not significant. However, the intervention condition × sex interaction was not significant in the time-specific incidence analysis. There were no adverse events.

**Table 4.** Empirical Distribution of Cumulative Incidence of Self-reported Forced Sexual Intercourse Perpetration and Experiences by Sex of Participants and Assessment Period, Mdantsane and Berlin, South Africa, 2004 to 2010

Assessment Period, mo	No./Total No. (%)		Boys vs Girls, RR (95% CI)	*P* Value	Intervention × Sex Interaction, RR (95% CI)^a^	*P* Value
	Boys	Girls				
Perpetrating forced sex						
Baseline	4/499 (1)	1/558 (0)	0.994 (0.986-1.002)	.16		
3	26/495 (5)	3/557 (1)	0.956 (0.938-0.975)	<.001	1.053 (1.013-1.095)	.01
6	47/495 (9)	5/557 (1)	0.923 (0.900-0.946)	<.001	1.065 (1.013-1.119)	.01
12	58/495 (12)	5/557 (1)	0.904 (0.881-0.928)	<.001	1.076 (1.021-1.134)	.006
42	78/495 (16)	19/557 (3)	0.894 (0.867-0.923)	<.001	1.063 (0.999-1.131)	.06
54	95/495 (19)	25/557 (4)	0.878 (0.849-0.907)	<.001	1.064 (0.996-1.138)	.07
3-54			0.968 (0.960-0.977)	<.001	1.019 (1.001-1.037)	.04
Experiencing forced sex						
Baseline	5/499 (1)	2/558 (0)	0.994 (0.984-1.003)	.21		
3	32/494 (6)	2/556 (0)	0.943 (0.924-0.963)	<.001	1.049 (1.006-1.094)	.02
6	51/494 (10)	4/556 (1)	0.914 (0.891-0.937)	<.001	1.061 (1.009-1.115)	.02
12	72/494 (15)	5/556 (1)	0.882 (0.857-0.907)	<.001	1.075 (1.017-1.137)	.01
42	91/494 (18)	26/556 (5)	0.885 (0.856-0.915)	<.001	1.060 (0.992-1.133)	.09
54	113/494 (23)	41/556 (7)	0.875 (0.844-0.907)	<.001	1.051 (0.978-1.131)	.18
3-54	NA	NA	0.965 (0.956-0.975)	<.001	1.017 (0.996-1.037)	.11

Abbreviations: NA, not applicable; RR, risk ratio.

^a^ Sex differences were analyzed with generalized estimating equation Poisson regression models adjusted for effects of the intervention and clustering of students within schools. Intervention × sex interactions were tested by adding the intervention × sex intervention term to the model testing sex differences.

## Discussion

The results indicated that the intervention reduced self-reported forced sex perpetration throughout the postintervention follow-up period. The adolescents randomized to the HIV/STD risk-reduction intervention were less likely to report forcing someone to have sexual intercourse 3, 6, 12, 42, and 54 months postintervention as compared with their counterparts in the attention-matched control condition. Boys were more likely to report perpetrating forced sex than girls, and the intervention was more efficacious in reducing forced sex perpetration among boys than girls.

The intervention also showed some promise in reducing forced sex experiences, particularly among boys. Although the intervention effect on incidence over the 54-month period was not significant, the HIV/STD risk-reduction intervention participants were less likely to report experiencing forced sex at 12 and 42 months postintervention compared with the control group. The reduction in the intervention group at 3, 6, and 54 months postintervention was not significantly different from the control group in the sample as a whole. However, significant interactions with sex indicated that at 3- and 6-month follow-up, the intervention reduced forced sex experience among boys.

It is perhaps not surprising that the intervention generally had more reliable effects on forced sex perpetration than experiences. Perpetration of forced sex is more within the volitional control of the individual than is experiencing forced sex. People can choose to force another person to have sex or to respect the person’s wish to say no to sex. In contrast, avoiding being forced to have sex is less within the volitional control of the individual. There are steps that individuals can take to reduce risk, and the intervention was designed to give participants the requisite knowledge and skills, but eliminating risk is substantially more difficult, especially when faced with a determined perpetrator.

It is notable that boys were more likely to experience forced sex than were girls, a finding observed in other studies in southern Africa.^3^ Although our data do not address the reasons for this, speculation might include the fact that young adolescent girls’ bodies are more protected owing to fear of pregnancy and HIV and concern about the family’s ability to collect lobola (ie, bride price, property [often cattle] a prospective husband gives to the head of the family of the prospective wife) on her marriage. There is also likely blindness to the sexual abuse of young adolescent boys, accompanied by the belief that males are not vulnerable to sexual assault.^5,43^

Two other studies hint at the possibility of reducing sexual assault among South Africans with behavioral interventions. However, both focused on IPV rather than forced sex more generally, which can be perpetrated on or by a broader range of people, including not only a romantic partner but also a family member, a stranger, or an acquaintance. A cluster RCT^44^ that combined a microfinance program and content on gender roles, domestic violence, and sex and HIV showed reduced IPV at 12-month follow-up. However, the outcome combined physical and sexual violence, making assessment of the intervention’s effects on sexual violence independent of physical violence impossible, and the participants were not adolescents, but adult women aged 33 to 49 years. Another cluster RCT^45^ reported that an intervention produced nearly significant (*P* = .05) reductions in perpetration of physical or sexual IPV by men; again, physical and sexual violence were combined and most participants (63%) were aged 18 years or older. Thus, to our knowledge, this RCT is the first to find that an intervention reduced the perpetration and experience of forced sex in South African young adolescent boys and girls.

The strengths of this study include the attention-matched control group, random selection of schools, high retention rates at long-term follow-up (with over 99% returning for at least 1 follow-up, including over 92% who returned 4.5 years postintervention), and the cluster RCT design, which increased internal validity while decreasing risk of contamination between groups. Another strength is that we assessed sexual coercion as a separate outcome, not combined with physical abuse as has been done in other studies. Worldwide, many studies have been faced with methodological challenges in assessing sexual coercion as an outcome, given its relatively lower prevalence in comparison with other forms of partner violence. In part, we could detect intervention effects in our sample because the rates of both perpetration and experiences were high.

### Limitations

The use of self-reports is a limitation common to all studies of forced sex. Our measure did not clarify the context including the perpetrators of forced sex experience and did not assess other forms of psychological abuse, unwanted kissing or touching, and threatening behaviors, information valuable to designing future interventions. However, our measures of forced sex were like those used in several studies examining forced sex in adolescents, and our findings of high reports of forced sexual experience among boys is consistent with other studies in southern Africa. The magnitude of the intervention effects in this trial was modest, about a 3% reduction in risk. Future research building on the present findings might incorporate booster sessions with additional activities focused on forced sex, which might enhance the intervention’s efficacy. Another limitation is that the intervention effects may not generalize to the larger population of South African adolescents or to implementation by teachers in classrooms across South Africa as opposed to the specially selected and trained facilitators who implemented the intervention in the trial.

## Conclusions

To our knowledge, this is the first large-scale community-level randomized intervention trial to show significant effects on forced sex among South African adolescents in the earliest stages of entry into sexual activity. The results suggest that intervening early, before sexual debut, can have long-lasting effects on forced sex. Indeed, the fact that adolescents who received only 12 hours of intervention in sixth grade, when few reported sexual experience, were less likely to report perpetrating and experiencing forced sex long after the intervention is extraordinary. Future implementation research must determine whether the characteristics of the intervention and its effects can be maintained with implementation in the real world. In addition, future research must identify the causal pathway that accounts for the intervention’s efficacy in reducing forced sex perpetration and experiences. Research along these lines is an important next step in addressing the worldwide public health problem of sexual assault.

## Notes

Supplement. **Trial Protocol**
