# Error in Byline Order

**DOI:** 10.1001/jamanetworkopen.2018.4304

**Published:** 2018-10-12

In the Original Investigation titled “Effect of a Behavioral Intervention on Perpetrating and Experiencing Forced Sex Among South African Adolescents: A Secondary Analysis of a Cluster Randomized Trial,”^1^ published August 17, 2018, there was an error in the byline order. Ann O’Leary, PhD, should have been listed as the second author and Loretta Sweet Jemmott, PhD, RN, as the third author. This article has been corrected.^1^
