# Supplementary material for: Effect of a Behavioral Intervention on Perpetrating and Experiencing Forced Sex Among South African Adolescents: A Secondary Analysis of a Cluster Randomized Trial
Source: JAMA Netw Open. 2018 Aug 17;1(4):e181213. doi: 10.1001/jamanetworkopen.2018.1213 (PMC6324287; doi:10.1001/jamanetworkopen.2018.1213)
Supplement: Supplement. — Trial Protocol [file jamanetwopen-1-e181213-s001.pdf]

University of Pennsylvania
Institutional Review Board (IRB) Application
Protocol Summary Outline

Date: May 4, 2009

Principal Investigator: John B. Jemmott III

Responsible Organization: Annenberg School for Communication

Protocol Title: South African Adolescent Health Promotion Project

Protocol #: 705388

---

1. Purpose

South African adolescents are at high risk for HIV infection because of unprotected sexual intercourse. What is needed is research to develop, implement, and test interventions to reduce South African adolescents' behaviors associated with this risk. Results of controlled studies in the US indicate that behavioral interventions, including the Be Proud! Be Responsible! curriculum, have reduced HIV sexual risk behavior among adolescents. Unfortunately, the available research literature contains few randomized controlled trials testing such interventions in sub-Saharan Africa and only one in South Africa.

The broad objective of the proposed research is to identify effective, sustainable interventions to dissuade South African adolescents from engaging in behaviors that increase their risk for sexually transmitted diseases (STDs), including HIV.

The participants will be 1,057 6th grade Xhosa speaking South African adolescents at 18 senior primary schools in Mdantsane and Berlin, Eastern Cape Province, South Africa. Matched pairs of schools will be randomly selected from among the senior primary schools that agree to participate in the study. The schools will be paired based on their similarity of the following factors: estimated number of 6th grade learners, number of classrooms with electrical outlets (a proxy for socioeconomic status), and the number of classrooms at each school. Additionally, rural and urban schools will be paired separately based on these criteria. One school in each pair selected will be randomly assigned to receive one of two interventions: (a) an HIV/STD risk-reduction intervention; or (b) a general health promotion intervention concerning health issues unrelated to sexual behavior, which will serve as the control condition.

The HIV/STD risk reduction intervention will be adapted from the Be Proud! Be Responsible! curricula found to be effective with African American adolescents in the U.S. This approach will draw on social cognitive theory, the theory of reasoned action/theory of planned behavior, and our previous risk-reduction research with inner-city African American adolescents.

Hypotheses. There are four hypotheses. They include the following:

- To examine whether the students in the HIV/STD intervention schools report less sexual risk behavior than do students in the control schools.
- To examine whether the intervention effects are significantly different among adolescents who vary in pre-intervention sexual experience, age, gender, intimate-partner violence, and sexual abuse history. This information would guide the development of future interventions tailored to subgroups of South African adolescents.
- To examine whether theoretically defined mediators of behavior change (that is, knowledge, behavioral beliefs, normative beliefs, perceived self-efficacy, and intentions) do indeed act as mediators of the intervention's effects on sexual risk behavior.
- To examine whether students in the HIV/STD intervention schools are less likely to test positive for a STD than are students in the control schools.

Statistical Power. Power analysis was performed to determine the sample size needed to detect effects of the interventions on self-reported frequency of unprotected sexual intercourse. We estimated the intracluster correlation coefficient from the pilot data we collected on 6th grade students at 4 schools in Mdantsane. The intracluster correlation for frequency of unprotected sexual intercourse was rho = 0.00864. Assuming alpha = .05, two-tailed, a total of 1,057 6th grade learners from the 18 schools in the trial with an average of 59 students in each school, the estimated power for tests of the effects of interventions on outcomes where the effect size is at least d = 0.25 is greater than 99% for attrition rates at the 12 month follow up of 15-20%.

We also estimated statistical power for STD incidence specifically. Some of the participants will be sexually experienced at the time of the follow-ups and therefore at risk of STD, but others will not be sexually experienced and therefore not at risk for STD. The power analysis for STD incidence must take into account the size of sample that is at risk. The median age of first sexual intercourse in South Africa is about 16 (MacPhail et al., 2002; Manzini, 2001; Richter et al., 2005; Simbayi et al., 2004). At baseline 76.54% of the adolescents in our study were 12 years or older and therefore will be 16 years or older at the 54-month follow-up. We considered three different rates of sexual experience and risk for STD at the 54-month follow-up: 76.5% (N=809), 51.3% (N=542), and 38.3% (N=405) of the original 1,057 participants.

Based on previous studies (Auvert et al., 2001; Johnson et al., 2005), we estimated that about 15.4% of the sexually experienced participants in the absence of a HIV/STD risk-reduction intervention would test positive for CT, GC, or TV at either the 42-month or the 54-month follow-up. We estimated power for detecting significant differences in STD incidence over the follow-up period for attrition rates of 15% and 20% at the 54-month follow-up, rho = 0.009, and STD incidence in the health-promotion control group = 0.154. At baseline, only 3.3% of participants were sexually experienced; hence, for the other 96% of participants, any STDs we detect will have been contracted during the follow-up period. The study is well powered to detect incidence differences in the range of at least 6.4% to 8.4%, depending on the true proportion of sexually experienced adolescents in the sample at the 54-month follow-up. Table 1 presents a range of estimates based on 38.3% to 76.5% of the baseline sample contributing to the STI incidence endpoint at the 54-month follow-up.

Table 1. Estimated power to detect a decrease in STD incidence assuming a cluster randomized controlled trial with a total of N=809, N=542, and N=405 sexually experienced participants enrolled from 18 matched pairs of schools (alpha = 0.05, two-tailed, rho = 0.009).

STD incidence			Attrition rate (54 months)					
			20%			15%		
Control	Intervention	Difference	N=809	N=542	N=405	N=809	N=542	N=405
0.154	0.10	0.054	0.76	0.58	0.47	0.78	0.61	0.49
0.154	0.09	0.064	0.88	0.73	0.60	0.90	0.75	0.63
0.154	0.08	0.074	0.95	0.84	0.72	0.96	0.86	0.74
0.154	0.07	0.084	0.98	0.91	0.81	0.99	0.93	0.84

Statistical Analyses. To address Hypothesis 1, we will use intervention type as the independent variable and model the incidence of sexual intercourse, unprotected sexual intercourse, and multiple partners as binary responses to test the effect of intervention using GEE on data collected preintervention and 3-, 6-, 12-, 42-, and 54-month follow-up.

Hypothesis 2 concerns whether the effects of HIV risk-reduction interventions may differ systematically among adolescents. Sexual experience, age, gender, intimate-partner violence, and sexual abuse history are the primary moderators of responsiveness to the HIV risk-reduction intervention we will consider. Although the notion that certain variables may moderate intervention effects is theoretically persuasive, little research has attempted to establish empirically the characteristics of intervention participants that might moderate the effectiveness of HIV risk-reduction interventions. Jemmott et al. (1998) found that a safer sex intervention was more efficacious with sexually inexperienced adolescents. We will test moderator effects using GEE, along the guidelines described by Baron and Kenny (1986) and Cohen and Cohen (1983). We will use GEE to test for significant interactions between intervention type and potential moderator, controlling for the main effects of all variables involved in the interaction. As an example, consider the moderation of intervention effects on intentions by age assessed as a continuous variable. By introducing an age-by-intervention interaction term, this moderation effect is easily tested using GEE.

Hypothesis 3 concerns mediation of intervention effects. The theoretical framework underlying the intervention suggests that knowledge, perceived self-efficacy, behavioral beliefs, normative beliefs, and intentions should mediate the effects of the intervention on sexual-risk behavior. Mediation will be tested using a standard procedure described by Baron and Kenny (1986). To establish mediation, it is necessary to show that (a) the independent variable affects the dependent variable; (b) the independent variable affects the putative mediator; and (c) the mediator has a significant effect on the dependent variable when the independent variable is controlled. Within our context, the analyses will proceed as follows: we will test for statistical significance of the intervention's effect on sexual behavior. We will then test the effect on the potential mediators. Finally, we will test whether the potential mediators have significant effects on sexual behavior, controlling for intervention effects. This set of analyses will indicate whether the 3 criteria of mediation are met. If the intervention effect on sexual behavior outcomes is nonsignificant, these analyses would provide valuable information as to whether a

lack of effects on mediators may account for it. In addition, the analyses would establish whether the changes in the mediators predict reduced risk behavior, even if the intervention effects are nonsignificant. In this way, it will be possible not only to gain insight into the underlying mechanisms of behavior change, but also to test the theoretical framework that was used to guide the creation of the interventions.

To address Hypothesis 4, we will use intervention type as the independent variable and model the incidence of STD as binary response to test the effect of intervention using GEE on data collected at the 42- and 54-month follow-up.

2. Duration

Funded by the National Institute of Mental Health
Start date: April 1, 2002
End date: August 31, 2010

3. Subject Recruitment and Selection

The participants will be 1,057 6th grade Xhosa speaking South African adolescents recruited from 18 senior primary schools in Mdantsane and Berlin, Eastern Cape Province, South Africa. About 59 participants will be recruited from each of the 18 schools. On average, participants will range in age from 11 years to 14 years at baseline. About one-half of the adolescents will be female.

Participants will be recruited by announcement in school classrooms. They will be invited to participate in the "South Africa Health Promotion Project." They will be told that the program is designed to understand South African adolescents' behaviors that may create health risks for heart disease, diabetes, cancer, unplanned pregnancy and STDs, especially HIV, and to find ways to teach South African adolescents how to avoid these dangers and risks.

In addition to making announcements to the students, we will present the study at parent-teacher association (PTA) meetings and address questions that parents or teachers may have. Xhosa speaking project staff will also be available to meet with parents when they pick up their child's report cards at the schools. If a parent does not attend the PTA meetings or the report card sessions, project staff will send invitations to their home to invite them to arrange for a meeting.

Some parents may be illiterate. In these instances, project staff will review the study and consent form with the parent orally and obtain their informed consent.

<u>Participation from Baseline to 12-month Follow-up</u>. Adolescents will be told that on 5 occasions, over the course of 12 months, they will complete a questionnaire. The adolescents will be told that the questions are very sensitive and personal, including questions about different sexual activities in which they may have engaged, the use of drugs and alcohol, the use of birth control, and whether they have had any previous STDs. They will be told that they will participate in small group activities, including discussions, games, and exercises. The risks and benefits of participation in the study will be explained.

The participants will receive various gift incentives for participating in the program over the 12 month period. After completing the initial intervention, participants will receive a notebook and pen. At the 3-month follow-up, they will receive a composition notebook and set of pens. At the 6-month follow-up they will receive a T-shirt with the project logo. Finally, at the 12-month follow-up they will receive a backpack with the project logo.

Adolescents must sign an assent form. In addition, their parent(s)/guardian(s) must sign an informed consent in order for adolescents to participate.

Copies of the assent form, consent form, and cover letter are included.

Participation in Long-Term Follow-up. The participants will be told that the objective of the long-term follow-up continuation of the study is to determine the long-term efficacy of the interventions at 42 and 54 months after participation. They will be told that on two occasions over the course of two years they will complete questionnaires and provide urine and blood samples. They will be told that the questionnaires are similar to the questionnaires they previously completed in the study. They contain profoundly sensitive and personal questions, including personal questions about different sexual activities in which they may have engaged; drug, alcohol or birth control use; and previous STDs. They will be told that their answers will be confidential, that they will be given a code number, and that the data will be organized by their code number, not their name.

The participants will be told that the urine sample will be tested for three (3) STDs: chlamydia, gonorrhea, and trichomonas and that the blood sample will be tested for HSV-2. Participants will be informed (a) that STDs are commonly asymptomatic, (b) that if they test positive for an STD, they will be given single-dose treatment free of charge (c) that their parents will not be informed of the test results and (d) that they can choose not to provide blood and/or urine specimen(s). If the participant does not provide blood and/or urine specimen(s), they will remain in the survey room with the data collector while the other participants are giving their specimens. The risk and benefits of participation in the study will be explained. All participants will be told that participation is voluntary.

We will contact participants to schedule cohort-specific meetings with them and their parents or guardians 5 weeks before each follow-up. The meetings will be held at the primary school where the interventions were held. Where this is not possible, the meetings will be held at the high schools where the participants are currently schooling. During this meeting we will distribute the parent/guardian consent form and a cover letter asking that the parents or guardians give their children permission to participate in the long-term follow-up study. The letter will ask the parent/guardian to read and sign the consent form and to give it to the child to return to project staff or to personally return it to project staff. The consent form and letter will be read aloud at the meeting, and the project staff will answer all questions and concerns presented. The parents will have the opportunity to complete the consent forms at the meeting. In cases where parents or guardians do not attend the meeting with the participants, the letters will be sent home with the participants. If the parents/guardians who did not attend the meeting cannot read, the Site Coordinator will read the introductory letter and consent form to them and will obtain their

informed consent. Within 5 days of the meeting, the Site Coordinator will return to the school to receive the signed consent forms and also address any other concerns or questions. The date of the return visit will be included in the letter that introduces the project. Should any participants or their parents or guardians fail to attend the meetings, the Site Coordinator will contact them by phone to arrange times to meet with them.

To facilitate the learners' attendance at the 42- and 54-month follow-up data-collection sessions, we will provide transportation for them to attend the sessions. In addition, about 3 weeks before each follow-up, we will remind the participants of their appointment, and we will remind them again 7 to 10 days before each follow-up. As in the initial funding period, the learners will be compensated with small gifts for participating. After completing the 42-month follow-up sessions, the boys will receive a cap and the girls will receive an umbrella, each imprinted with the study logo. At the 54-month follow-up, all participants will receive a jacket with the study logo. We will invite those who fail to attend the scheduled follow-up session to complete the questionnaire during make-up sessions at the project offices at the University of Fort Hare, their school, or a convenient community location (e.g., public library, the school at which they originally participated).

The participants must sign assent forms in addition to their parents/guardians signing informed consent forms for the adolescents to participate. Before the adolescents complete the follow-up questionnaire, the data collector will use a standard script to reiterate the description of the study. Participants will be told that they are volunteering to be in the study and that they can elect not to participate and can withdraw at any time.

We will not reimburse the participants monetarily because our South African collaborators and advisers did not believe that would be appropriate. Participant reimbursements were set at a rate that would not constitute coercion.

4. Location

The intervention sessions and preintervention, postintervention, and 3-, 6-, and 12-month follow-up sessions will be held at 18 schools in Mdantsane and Berlin, South Africa during school hours over a period of 13 months. These intervention and data-collection sessions will be held during the extracurricular activity period that is held each afternoon. The 42-month and 54-month data collection sessions will be held at Fanti Gaqa Senior Primary School located in Zone 6 of Mdantsane, central to all schools participating in the project.

5. Background

As of December 2000, 70% of the world's 36 million people with HIV/AIDS live in sub-Saharan Africa (UNAIDS, 2000b). South Africa has the largest number of persons living with HIV/AIDS in the world, an estimated 4,200,000 people, and the number of new cases in South Africa is increasing at an alarming rate. The current estimated prevalence of 20% of the adult population is a dramatic increase from the 13% prevalence in 1997 (UNAIDS, 2000b).

Unlike other parts of the world, in sub-Saharan Africa, including South Africa, more women than men are infected with HIV and dying of AIDS, and heterosexual contact is the chief mode of HIV transmission. Unfortunately, efforts at behavioral prevention have been hampered because few controlled studies have been conducted to identify the most effective ways to change risk behavior in the sub-Saharan Africa context (Gouws & Williams, 2000; Harrison et al, 2000). The proposed study is in response to this problem.

This research proposal focuses on sexual risk reduction among Xhosa-speaking adolescents in Eastern Cape Province, South Africa. These young people are at great risk of developing HIV infection. National surveillance surveys of pregnant women attending antenatal clinics revealed that those less than 20 years of age had an HIV prevalence of 21% in 1998 (Department of Health, 1998a). This is a dramatic increase from the 2% prevalence in this age group in 1991(UNAIDS, 2000c).

Young adolescents are also at high risk. More than 40% of South Africans are under 15 years of age, and 10% of antenatal clinic women in this age group tested positive for HIV. At the current rate of infection, it is estimated that more than 50% of South Africans under 15 years of age today will die of AIDS in the next 10 years (UNAIDS, 2000b). In the Eastern Cape, 14% of antenatal women under 20 years of age have tested positive for HIV (Meidany & Puchert, 2001).

STD infection rates among South African adolescents may place them at increased rates for sexually transmitted HIV infection. Although there is no national surveillance system for STDs in South Africa, a literature review estimated that 15% of family planning and antenatal clinic attendees were seropositive for syphilis, 16% had chlamydia (CT), and 8% had gonorrhea ([GC] Pham-Kanter et al, 1996). Johnson et al.'s (2005) recent review of 47 independent studies also revealed high STD prevalence among women attending antenatal and family planning clinics. The median estimated prevalence was 10% for syphilis, 11% for CT, 5% for GC, and over 20% for Trichomonas vaginalis (TV). STDs can increase the efficiency of sexual transmission of HIV (Fleming & Wasserheit, 1999; Grosskurth et al., 2000). Consistent with this, Pettifor et al.'s (2005) national survey of South Africans 15 to 24 years of age found that a history of STD symptoms was associated with HIV infection, and Auvert, Ballard et al. (2001) tied biologically confirmed STDs to positive tests for HIV.

As in the US, STDs affect young people disproportionately. It is estimated that 4 million STD cases occur each year in South Africa, with over half of these infections occurring among adolescents and young adults (Dickson-Tettech & Ladha, 2000). Data from the 2000 antenatal clinic survey in the Eastern Cape revealed that young people 24 years of age and younger had the highest syphilis seropositive rate of any age group (Meidany & Puchert, 2001).

There are no national data on rates of CT, GC, or TV among South Africans 15 to 16 years of age. However, Dr. Ron Ballard (2007) of the CDC has provided relevant data from a study by Auvert, Ballard et al. (2001) on youth in a township in the Carletonville district of the Gauteng Province, South Africa. That population-based, cross-sectional study used random sampling techniques to select households. Urine specimens were tested for CT and GC using ligase chain reaction assays. Although the article did not report results for adolescents 14 to 19 years of age separately, Ballard provided us with the rates of these STDs in this sub-sample of 304 girls and

299 boys. The prevalence of CT was 9.9% and 3.7% in girls and boys, respectively. The prevalence of GC was 7.2% and 1.3% in girls and boys, respectively.

Statistics on adolescent pregnancy also provide evidence that South African adolescents are at risk for sexually transmitted HIV infection (Jewkes et al, 2000b; Makiwane, 1998). In 1998, 16.8% of adolescent women 15 to 19 years of age were mothers or pregnant (UNAIDS, 2000c). By the age of 19 years, 35% of all South African adolescents have been pregnant or have had a child (Department of Health, 1998b).

The best way for adolescents to avoid sexually transmitted HIV infection is to avoid sexual activity. Data from a recent nationally representative random survey of 2,000 South African adolescents 12 to 17 years of age revealed early onset of sexual activity (Kaiser Family Foundation, 2001). About 31% of the respondents reported having had sexual intercourse, 18% of the sexually experienced adolescents reported having their first sexual experience at age 12 or younger, and 78% reported their first experience by age 15. In addition, about 18% of sexually experienced South African adolescents report currently having multiple partners, and among sexually experienced boys, the figure is 26%.

There is also evidence that South African adolescents fail to use condoms consistently. About 41% of sexually experienced adolescents say they do not always use condoms (Kaiser Family Foundation, 2001). Data on contraception use suggested that Black South African adolescents (42%) were less likely to report using contraception than were their White (63%), Indian (48%), and Coloured (61%) counterparts. In addition, younger adolescents 12 to 13 years of age were less likely to report using contraception than were older adolescents, 16 to 17 years of age. Our survey of Xhosa-speaking 6th graders revealed that only 32% reported using a condom the first time they had sexual intercourse and only 27% reported using one during their most recent sexual intercourse experience (Jemmott et al., 2001).

In summary, South African adolescents are at high risk for HIV infection because of unprotected sexual intercourse. What is needed is research to develop, implement, and test interventions to reduce South African adolescents' behaviors associated with this risk. Although there is a consensus that HIV/STD behavioral interventions can be effective (Interventions to Prevent HIV Risk Behaviors, 1997), questions about the generalizability of intervention effects across different populations and cultural contexts remain unanswered. Most HIV/STD prevention interventions have been implemented and tested with only one or two populations; hence, their replicability and generalizability have yet to be demonstrated (Auerbach & Coates, 2000). Despite the urgent need for HIV/STD prevention interventions for South African adolescents, only one study has rigorously evaluated such an intervention (Harvey et al, 2000). Reviews of the literature have urgently called for research to examine the generalizability of successful and effective HIV/STD prevention interventions (Auerbach & Coates, 2000) and, more specifically, to identify evidenced-based HIV/STD behavioral interventions for South Africans (Gouws & Williams, 2000; Harrison et al, 2000). This proposal is in response to those calls.

6. Research Design

Prior to conducting the interventions, we will conduct formative research including focus groups, elicitation surveys, and pilot studies to refine the procedures, the study instrument, and the interventions. The focus groups will include adolescents and parents of adolescents.

Adolescent Focus Groups. We plan to conduct 6 focus groups with Xhosa-speaking 6th graders (i.e., about 12 years of age): 2 all-male groups, 2 all-female groups, and 2 mixed-gender groups. Each group will include 10 adolescents. The single-gender groups will be led by same-gender co-facilitator pairs; the mixed-gender groups, by male and female facilitator pairs. The groups will be conducted in Xhosa. We will train Xhosa-speaking graduate students who are closer in age to the participants to run some groups. The adolescent focus groups will help us to accomplish several goals.

Parent/Guardian Focus Groups. We will conduct 3 focus groups with parents or guardians of adolescents: an all-female group, an all-male group, and a mixed-gender group. Each group will involve approximately 10 participants. The purpose is to solicit parent/guardians' input regarding the adolescents' behaviors we seek to change.

Pilot Testing the Questionnaire and Interventions. We will pilot test the revised questionnaire with 20 6th grade students in Mdantsane. We will then pilot test the interventions with 30 6th grade adolescents at a school in Mdantsane. One-half of the students will be randomly assigned to receive the HIV/STD sexual risk reduction intervention and, the others, to receive the general health-promotion control intervention.

The pilot participants will complete the pre-intervention questionnaire and then receive their intervention in 6 2-hour weekly sessions. They will subsequently complete the post intervention questionnaire. We will debrief the participants to solicit their comments regarding how we might improve the project. We will use this experience to make final revisions to the intervention and questionnaires.

Intervention Design. The design of the study is a randomized controlled trial. The schools will be randomly assigned to receive either the HIV/STD risk-reduction intervention or the general health-promotion control intervention. The study will be held during the extracurricular activity period at 18 schools in Mdantsane and Berlin, South Africa. The intervention will be in conducted in Xhosa and will consist of 6 2-hour sessions.

On a day before the first session of the intervention, all adolescents will complete a Xhosa pre-intervention questionnaire measures. After completing the questionnaires, adolescents will attend the first intervention session, which will be implemented by specially trained Xhosa speaking male and female co-facilitators. The intervention will be implemented over 6 sessions.

Intervention Type has 2 levels:

- HIV/STD risk-reduction intervention consisting of 12 1-hour modules over 6 sessions,

- General health-promotion control intervention consisting of 12 1-hour modules over 6-sessions, which serves as a control group.

The interventions differ in content, but are structurally similar. Adolescents will participate in a small group with 10 to 16 adolescents led by two specially trained Xhosa speaking adult co-facilitators. Intervention activities include small group discussions, interactive exercises, brainstorming, games, and role-playing designed to increase knowledge, motivation, and skill related to the behaviors that are the focus of the particular intervention.

Immediately after the last intervention session, the participants will attend a session to complete the Xhosa post intervention questionnaire. All participants will be contacted 3, 6, 12, 42, and 54 months post intervention and invited to complete the Xhosa follow-up questionnaire.

<u>STD incidence</u>. As a secondary outcome, we will examine Chlamydia trachomatis (CT), Neisseria gonorrhea (GC), and Trichomonas vaginalis (TV) based on nucleic acid amplification tests on urine specimens and HSV-2 based on an ELISA test at 42-month and 54-month follow-ups. We will test the effects of the intervention on the presence of any of these four STDs at either the 42-month follow-up or the 54-month follow-up. We selected these STDs because of their high rates in this population and the availability of accurate tests for them. According to the World Health Organization (2001), CT is the most common bacterial cause of STD with an estimated 89 million annual cases worldwide, and GC is also common with over 60 million annual cases globally. However, the most common non-viral STD worldwide is caused by TV, with over 174 million estimated cases in 1999. Participants will go to the rest room and provide a first-void urine specimen of approximately 20 to 30 cc. Urine specimens will be refrigerated and delivered to the STI Reference Centre, National Institute for Communicable Diseases in Johannesburg within 28 days. A dedicated refrigerator at the project offices will be utilized for storing specimens exclusively before transporting them to the laboratory. A blood sample will be collected from each participant and shipped to the STI Reference Centre to be tested for herpes simplex 2 (HSV-2). David Lewis FRCP(UK) PhD, the Head of the STI Reference Centre, has agreed to serve as a co-investigator on the study to oversee the biospecimen collection, storage, shipping, and testing.

We will employ Gen-Probe's Aptima combo assay for CT and GC and Aptima trichomonas vaginalis assay for TV. The sensitivity of Gen-Probe's tests for CT and GC and TV has been shown to be superior to culture and direct specimen tests. The greater sensitivity of the tests permits the use of specimens other than endocervical swabs for women and urethral swabs for men. It is the only assay with urine specimen sensitivity equivalent to swab specimen. An enzyme-linked immunosorbent assay (ELISA) test will be used to detect HSV-2 type specific IgG antibodies.

Clinical research coordinators will be trained to handle the biological specimens. A 2-day training will familiarize them with the follow-up study, train them in the details of their responsibilities, and emphasize the importance of confidentiality. Blind to the participants' original intervention condition, they will collect and handle the biological specimens and provide pre- and post-test counseling to participants. Participants will be notified of their test results, and detected CT, GC, and TV infections will be treated according to CDC guidelines. For both

ethical and methodological reasons, it is imperative that participants receive complete treatment. We will, therefore, treat participants with directly observable state-of-the art single-dose oral therapy for these STDs to minimize potential non-adherence to multi-dose medication regimens.

Dr. Anthea Klopper MBChB (UCT), MRCGP DCH DA, a general practitioner and child development specialist who has a practice in East London, has agreed to serve as a Research Physician on the study to oversee the treatment of STDs and any follow-up care. She will be responsible for ensuring that those participants who test positive for chlamydia, gonorrhea, or trichomonas are treated according to CDC guidelines and that those who test positive for HSV-2 are referred for treatment and counseled. Dr. Klopper will monitor the work of the Research Nurse who will dispense the medication to the participants who test positive for chlamydia, gonorrhea, or trichomonas and will refer participants as appropriate for additional follow-up care. Dr. Klopper will order the medication that will be used to provide the single-dose treatment to STI positive participants as indicated in the protocol.

Attached are copies of the female and male versions of the pre-intervention questionnaire, which were previously reviewed and approved by the IRB Committee. Compared with the female version of the questionnaire, the male version has only minor differences in a few questions (e.g., gender pronouns, boyfriend vs. girlfriend). Also attached is the questionnaire that will be used at the 42-month and 54-month follow-up.

Amendment to Study the Impact of Father Involvement on Xhosa Adolescents. This exploratory research seeks to collect information on the involvement of fathers in promoting HIV/STD risk reduction in their adolescent children. If the preliminary research suggests that interventions to help fathers reduce the health risks of their adolescent children are feasible and acceptable, then we would pursue additional research on father-child sexual risk reduction interventions in South Africa. We plan to conduct 8 focus groups with adolescents and parents of adolescents. All focus groups will be conducted in Xhosa and will be single-gender (all boys, all girls, all fathers, or all mothers). Two of each type of group will be conducted. Each group will last about 2 hours and will have about 10-12 participants. The focus groups will explore beliefs about fathers, father absence, father-adolescent relationships and communication, father involvement, the impact of fathers, why fathers might be absent, and ways of coping with father absence. They will also explore the impact of other males, including grandfathers and older brothers. The participants will complete a brief pre-focus group questionnaire before participating in the focus groups so that we can characterize the people who participated.

In addition, we plan to conduct a survey with about 160 parent-adolescent dyads. This would include father-son dyads, father-daughter dyads, mother-son dyads, and mother-daughter dyads. About 40 of each type of dyad will be included in the study. The parents' survey will include measures of attitudes toward adolescent sexual involvement, attitudes toward parent-child communication, parent-child communication about sexual risks, self-efficacy regarding communicating with their child about sexual risks, quality of the parent-child relationship, parental monitoring and supervision. The adolescents' survey includes measures of attitudes, subjective norms, outcome expectancy, self-efficacy and intentions regarding sexual risk behaviors, self-reported sexual risk behaviors, father absence, father involvement, including communication and relationship with the child, and potential mediators and moderators of

relation between father absence and involvement and adolescents' sexual risk behavior, including abstinence, condom use, and unprotected sexual intercourse.

7. Potential Risks

There are mild psychological risks to the adolescents. The frank personal questions in the questionnaires may upset them. Unexpected sensitive and personal questions are apt to be more upsetting than such questions that people expect and for which they can prepare themselves. Hence, the risk of upsetting participants in this research with the sensitive and personal questions is minimized by the fact that participants agree to and will expect the questions.

We have used similar questionnaires in previous studies with 6th and 7th grade African American adolescents without any adverse reactions to the questions. In addition, we pilot tested a draft of the questionnaire with Xhosa speaking 6th grade adolescents and none were upset by the questions.

The participants will also be told that they can choose not to answer questions that they are uncomfortable answering. In our previous research, we have found that these procedures have effectively minimized anxiety and distress in response to the questions. Another issue that sometimes arises with questionnaires is discomfort around literacy and knowledge. Xhosa speaking data collectors and other Xhosa speaking staff members will all be trained to minimize and respond to participant discomfort.

Although the interventions are not designed to touch on the issue of sexual abusive experiences, there is the possibility that such experiences may be identified during the intervention. In our previous intervention studies, adolescents have not displayed distress over such an experience. However, should it occur we will follow the procedures that the schools would follow if school personnel suspected child abuse. Some schools have a CARE Team that is trained by the Social Welfare and Health Department to identify children who have been sexually or physically abused. If a teacher has reason to suspect that a child has been abused, the teacher informs the CARE Team or the principal. The case is reported to the area social worker. Whether sexual or physical the case would then be reported to the Child Protection Unit at the local police station. The social worker will bring in an appointed medical doctor and rehabilitative services as needed. Some of police stations in the Eastern Cape now have a victim support center (usually used by rape victims), and there are plans for special sections of courts to be set-aside for children's cases. There are also some safe houses in, but nothing like the number needed in light of the prevalence of child abuse and domestic violence.

8. Consent Procedures

A signed parent/guardian consent form is required for adolescents to participate. In addition, participants will sign an adolescent assent form. During recruitment sessions held in the adolescents' school, the Project Director and other project staff will describe the study and invite adolescents to participate. They will then distribute to potential adolescent participants parent/guardian consent forms and cover letters addressed to parent/guardians.

Project staff would return to the school to collect the signed parent/guardian consent forms at a later date before the initial intervention session. Before completing the pre-intervention questionnaire, the participants will be administered the adolescent assent form.

Copies of the parent/guardian consent form and the adolescent assent form are attached.

An additional signed parent/guardian informed consent form and an additional signed adolescent assent form will be required for participation in the long-term follow-up component of the study because this activity was not mentioned in the original consent and assent documents. The project staff will contact the participants, describe the follow-up study and invite adolescents to participate. They will schedule meetings at which the Project Director and the Site Coordinator will meet with participants and their parents/guardians. At these meetings the consent forms and letters will be distributed and read aloud and the follow-up study will be introduced. Where participants attend the meetings without their parents/guardians, they will be given the consent forms and introductory letters to take to their parents or guardians. In cases where the parents cannot read the consent form, the Site Coordinator will read the introductory letter of the study and consent form to them and will obtain their informed consent. The letter will ask the parent/guardian to read and sign the consent form and to give it to the child to return or to return it themselves to the school where the Site Coordinator will meet them on the date indicated in the letter. The Site Coordinator will address any additional questions or concerns on that date.

The consent form will provide sufficient information about the procedures and the risks and benefits of participation to give the parent or guardian a complete understanding and to permit informed consent. The information will be conveyed in a clear, easy to understand manner. A copy of the informed consent document will be given to the parent/guardian. They will also be provided with contact information for Investigators and IRB personnel who can answer questions that may arise in the future.

In addition to parent/guardian informed consent, the adolescents will read and sign an Adolescent Assent Form in order to participate in the long-term follow-up component of the study. Participants will be told that they are volunteering to be in the study and that they can elect not to participate or can withdraw at any time. The participants will read and sign the Assent Form. A copy of the assent document will be given to the participants. They will also be provided with contact information for Investigators and IRB personnel who can answer questions that may arise in the future.

Documentation of informed consent, including signed parent/guardian informed consent forms and adolescent assent forms, will be stored in a locked cabinet to which only authorized personnel have access.

9. Protection of Program Participants

The confidentiality of the information about participants will be maintained. No names or identifying information will be included in research reports. Participants' names will not appear on questionnaires. Participants' names will not appear on the biological specimens. Parents will not be informed of the results of the STD tests. Participants will be given code numbers and

information about them will be organized by code number--not name. The list of code numbers and names will be kept in a locked cabinet accessible only to authorized research staff.

We considered several factors in deciding against notifying parents of the adolescents' STD test results. If the adolescents believed that their parents would be told the results of their tests, the adolescents would be less likely to participate, which could bias the sample through self-selection. Parental notification does not concern protection of research participants, but parental rights. Adolescents who are tested have the opportunity to be treated for a potentially asymptomatic condition, whereas those who do not participate will not have this opportunity. This is a case where participation decreases risk.

10. Potential Benefits

Adolescents may learn about prevention of pregnancy and STDs, including HIV, or other major health risks of South Africans. They will learn ways to protect themselves from these health problems. They may enjoy participating since participants in similar projects have reported enjoying the interventions.

Adolescents will learn whether they have a STD, which may be asymptomatic. In South Africa, STD treatment is based on the presentation of symptoms, not screening. Thus, this study would provide a unique opportunity for the participants to have any asymptomatic STDs identified. STDs can cause pelvic inflammatory disease and infertility and are believed to be a co-factor in HIV transmission. They will receive treatment if they test positive. In addition, adolescents will be referred as appropriate for any mental health problems that become apparent during this research.

There are also benefits that may stem from the proposed research that transcend the individual participants. The participants will contribute to scientific knowledge that could be used to prevent these health problems in South African adolescents. STDs, including HIV, are important health problems affecting the South African community. Unfortunately, efforts to prevent these health problems are constrained by a dearth of hard data on the causes of high-risk behavior.

11. The Risk/Benefit Ratio

The risks of this research are relatively minimal. Considering probability and magnitude, they are not greater than are the risks ordinarily encountered in daily life or during the performance of routine physical and psychological examinations or tests. In contrast, the potential benefits are great. In short, the risks to the individual participants are reasonable in relation to the anticipated benefits to them as individuals and in relation to the importance of the scientific knowledge that will accrue from this research.

The risks of participation in the long-term follow-ups in this research are small relative to the potential benefits. South Africa has more cases of HIV/AIDS than any other country, and the incidence among young people under 20 years of age is extremely high. The risk to the individual participants is reasonable in relation to the anticipated benefits to them as individuals

and in relation to the importance of the scientific knowledge that will accrue from the long-term follow-up component of this research.
